# Investigating the molecular mechanisms associated with ulcerative colitis through the application of single-cell combined spatial transcriptome sequencing

**DOI:** 10.3389/fimmu.2025.1534768

**Published:** 2025-05-13

**Authors:** Hua Huang, Jiaze Ma, An Kang, Tianwei Guo, Wei Sun, Yan Xu, Lijiang Ji

**Affiliations:** ^1^ Department of Anorectal Surgery, Changshu Hospital Affiliated to Nanjing University of Chinese Medicine, Changshu, Jiangsu, China; ^2^ No. 1 Clinical Medical College, Nanjing University of Chinese Medicine, Nanjing, Jiangsu, China; ^3^ School of Pharmacy, Nanjing University of Chinese Medicine, Nanjing, Jiangsu, China; ^4^ Department of Pathology, Changshu Hospital Affiliated to Nanjing University of Chinese Medicine, Changshu, Jiangsu, China; ^5^ Department of Oncology, Changshu Hospital Affiliated to Nanjing University of Chinese Medicine, Changshu, Jiangsu, China; ^6^ Department of Pharmacy, Affiliated Changshu Hospital of Nantong University, Changshu No. 2 People’s Hospital, Changshu, Jiangsu, China

**Keywords:** immune infiltration, single-cell transcriptome sequencing, spatial transcriptome sequencing, ulcerative colitis, T cell exhaustion

## Abstract

**Background:**

Ulcerative colitis (UC) is a chronic inflammatory bowel disease marked by dysregulated immune responses, resulting in sustained inflammation and ulceration of the colonic and rectal mucosa. To elucidate the cellular subtypes and gene expression profiles implicated in the pathogenesis of UC, we utilized single-cell and spatial transcriptomic analyses.

**Methods:**

We conducted an analysis of single-cell data to identify cell types involved in the pathogenesis of UC. Employing machine learning methodologies, we screened for key genes implicated in UC and validated these findings through spatial transcriptomics. Additionally, immunohistochemistry was performed on UC lesion samples to investigate the expression patterns of the identified key genes. In an animal model, we utilized immunofluorescence and western blotting to validate the expression of these genes in the affected intestinal segments.

**Results:**

Our investigation identified specific monocyte subtypes associated with UC through a comprehensive analysis involving cell communication, Least Absolute Shrinkage and Selection Operator (LASSO), and Support Vector Machine (SVM) methodologies. Notably, two genes, G protein subunit gamma 5 (*GNG5*) and tissue inhibitor of metalloproteinase 1 (*TIMP1*), were identified as key regulators of UC development. Spatial transcriptomic indicated a downregulation of *GNG5* expression in UC, whereas *TIMP1* expression was upregulated. Furthermore, a significant correlation was detected between *TIMP1* and T cell exhaustion-related genes such as genes related to T cell exhaustion, including T cell immunoreceptor with Ig and ITIM domains (*TIGIT*) and cytotoxic T-lymphocyte-associated protein 4 (*CTLA4*). Immunohistochemical analysis of UC lesion samples revealed diminished expression levels of *GNG5* and elevated expression levels of *TIMP1*. A dextran sulfate sodium (DSS)-induced colitis mouse model was developed, demonstrating that the protein expression levels of *GNG5* in the colonic tissue of model mice were significantly decreased compared to controls w)ile the expression levels of *TIMP1* were increased (*p* < 0.01). Furthermore, immunofluorescence staining indicated co-localization of *TIMP1* with the macrophage marker F4/80 in monocytes.

**Conclusion:**

Our research delineated distinct monocyte subtypes correlated with UC and identified two pivotal genes, *GNG5* and *TIMP1*, that contribute to the disease’s pathogenesis. These insights offer a significant theoretical basis for enhancing the clinical diagnosis and therapeutic strategies for patients with UC.

## 1 Background

Ulcerative colitis (UC) is a chronic, idiopathic form of inflammatory bowel disease (IBD) that predominantly affects the mucosal and submucosal layers of the colorectal region. The pathogenesis of UC is characterized by dysregulated immune responses, resulting in persistent inflammation and ulceration of the colonic and rectal mucosa. Contributing factors include genetic predisposition, environmental influences—including infections and dietary components—and an exaggerated immune response to gut microbiota. These factors collectively undermine the integrity of the mucosal barrier, facilitate the infiltration of inflammatory cells, and promote the release of pro-inflammatory mediators. Epidemiological evidence suggests that UC is relatively prevalent in developed countries, with high incidence rates in North America and Europe (1). In recent years, however, there has been an observable increase in the incidence of UC in many newly industrialized countries, including China, coinciding with global economic development and dietary changes (2). This trend is particularly concerning given the generally reduced life expectancy of UC patients, alongside their heightened risk of requiring colectomy and progression to colorectal cancer. Therefore, the active investigation of UC pathogenesis and the formulation of precise therapeutic strategies have become urgent research imperatives.

Currently, the management of UC primarily involves the administration of 5-aminosalicylic acid (5-ASA) preparations and glucocorticoids. While these pharmacological agents frequently offer prompt alleviation of symptoms, they are also linked to considerable toxic side effects and low patient adherence. Immunosuppressants are primarily employed for maintenance therapy following the remission of symptoms induced by glucocorticoids, with the objective of minimizing glucocorticoid dosage. Additionally, biological agents, specifically monoclonal antibodies that target distinct inflammatory mediators such as tumor necrosis factor or integrins, are incorporated into the therapeutic regimen. The American Gastroenterological Association (AGA) guidelines (3) advocate for the initiation of biologic therapy as a first-line treatment and suggest early step-down strategies, thereby surpassing traditional treatment approaches (4). In the context of selecting biologics for UC, current clinical guidelines endorse the use of vedolizumab (VDZ) or anti-tumor necrosis factor alpha (TNF-α) agents (5). It is important to highlight that over 30% of patients demonstrate resistance to TNF-α therapies, with a subset eventually necessitating intestinal or colon resection surgery (6). In China, over 50% of patients with IBD show suboptimal responses to treatment after approximately one year of first-line anti-TNF-α therapy (7). This secondary dysregulation may be attributed to the immunogenicity of TNF-α antibodies, leading to the development of drug-resistant antibodies (8). Therefore, a deeper investigation into the intricate biological mechanisms underlying UC is essential for advancing the development of effective therapeutic strategies.

Single-cell transcriptome sequencing (scRNA-seq) is a sophisticated technique employed to examine RNA expression at the individual cell level, revealing cellular heterogeneity and the transcriptional profiles of specific cell types. Our preliminary single-cell analysis revealed significant increases in the populations of Plasma cells, activated memory CD4^+^ T cells, resting Natural Killer (NK) cells, M0 Macrophages, M1 Macrophages, activated Dendritic cells, activated Mast cells, and Neutrophils in patients with UC compared to healthy controls. Subsequently, we conducted an in-depth investigation into the expression of target genes within immune cells, taking into account the complex interactions between bile acid metabolism and immune cell dynamics (9). Spatial transcriptome sequencing, which retains the spatial context of tissues, quantifies gene expression through methods such as microarrays applied to tissue sections or spatial fluorescence *in situ* hybridization. This methodology facilitates the examination of gene expression within specific tissue regions. The integration of single-cell and spatial transcriptome sequencing techniques permits the concurrent exploration of mechanisms at both the cellular and tissue levels, providing novel insights into the complex mechanisms underlying diseases (10). In this study, we integrated single-cell and spatial transcriptome sequencing to identify target genes associated with UC and conducted a preliminary investigation into the interrelationships among these target genes, immune cells, and the microenvironment. This approach was designed to advance our understanding of UC therapeutic targets and the underlying mechanisms.

## 2 Materials and methods

### 2.1 Study design

**Figure 1:**
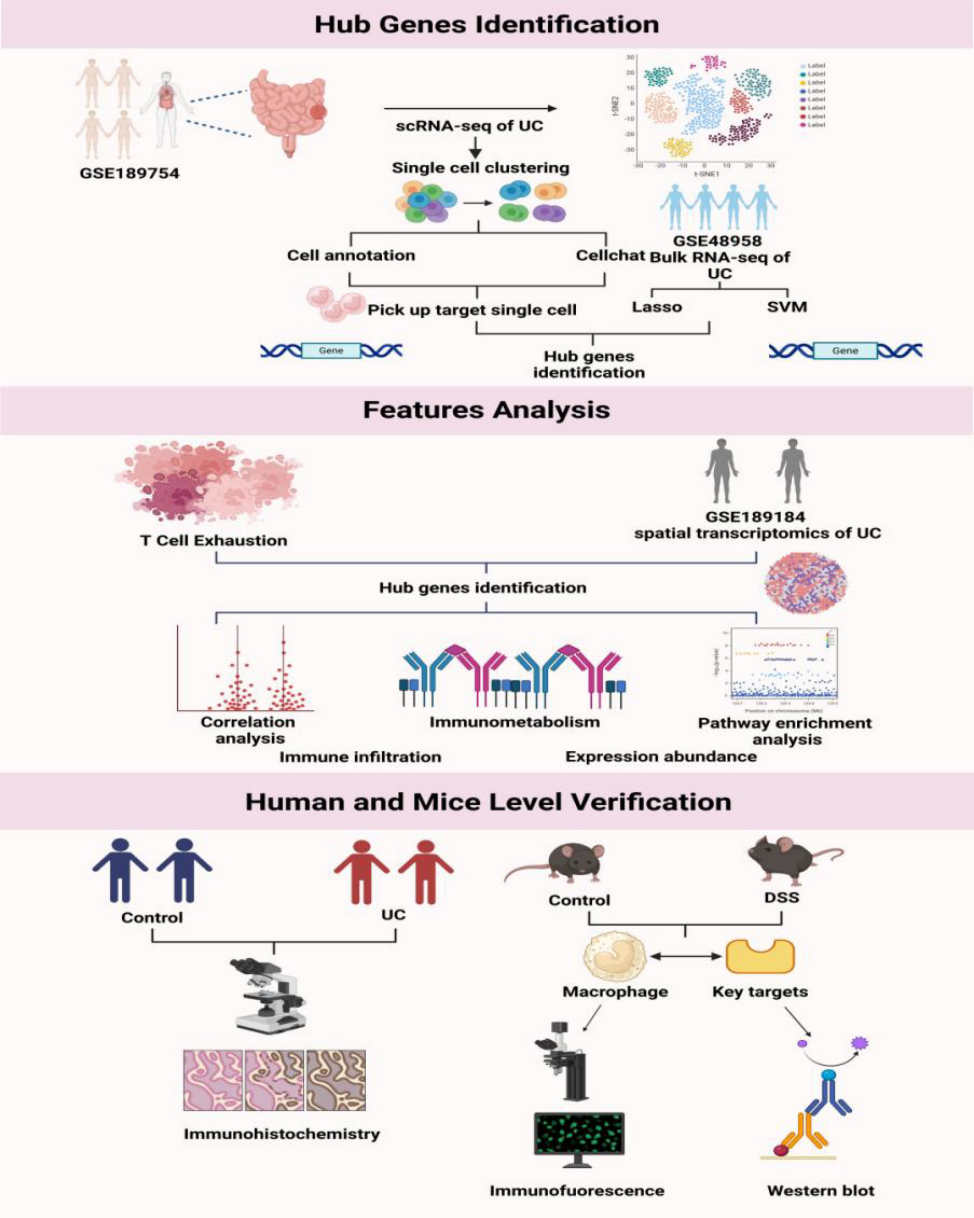
Research flowchart.

The study design is presented in **Figure 1**.

### 2.2 Data acquisition

The Gene Expression Omnibus (GEO) database (https://www.ncbi.nlm.nih.gov/geo/info/datasets.html), curated by the National Center for Biotechnology Information (NCBI), functions as a comprehensive repository for gene expression data. From this resource, we acquired the single-cell transcriptome data corresponding to GSE189754, concentrating on 11 samples that provided complete single-cell expression profiles for single-cell analysis. Additionally, we downloaded the spatial transcriptome data for GSE189184, selecting two control groups (B10, C5) and two disease groups (B8, B4) for analysis. Furthermore, we procured the transcriptome data for GSE48958, encompassing data from 8 controls and 13 disease samples.

### 2.3 Quality control and data standardization

In this study, the processes of quality control and data standardization are essential to ensure the accuracy of subsequent analyses. We employed the Seurat package (11) for initial data processing. For cell quality control, we conducted screening based on the total number of unique molecular identifiers (UMIs) per cell, the number of expressed genes, and the proportion of mitochondrial gene expression. Typically, a high proportion of mitochondrial gene expression in a cell indicates low RNA expression levels and potential progression towards cell death, warranting the exclusion of such cells. Additionally, we utilized the median absolute deviation (MAD) for quality control, removing outliers that deviate from the median by more than three times the MAD to maintain data reliability. Subsequently, we applied DoubletFinder (version 2.0.4) (12) to individually filter doublet cells in each sample, thereby completing the comprehensive cell quality control process.

In the data standardization process, the LogNormalize method of global normalization is employed. This technique mitigates the impact of variations in total RNA content between cells on gene expression analysis by scaling the total expression level of each cell with a coefficient \(s_0\), adjusting it to 10,000, and subsequently normalizing it through logarithmic transformation. Cell cycle scores are computed using the CellCycleScoring function, and highly variable genes are identified via the FindVariableFeatures function. The ScaleData function is utilized to eliminate gene expression fluctuations attributable to mitochondrial gene expression, ribosomal gene expression ratios, and cell cycle differences. Linear dimensionality reduction is conducted on the expression matrix using the RunPCA function, with 20 principal components selected for further analysis. The Harmony algorithm is applied with default parameters to correct for batch effects, and finally, the RunUMAP function is employed with default parameters for nonlinear dimensionality reduction.

### 2.4 Identification of cell clusters

Cell types and corresponding marker genes were identified using CellMarker (13), PanglaoDB (14), and literature, supplemented by automated annotation with SingleR (15) software. The FindAllMarkers function was employed to filter marker genes within each category, with only positive markers expressed in at least 25% of the cells retained (only.pos = TRUE, min.pct = 0.25).

### 2.5 Ligand receptor interaction analysis (Cellchat)

CellChat (16) is a sophisticated tool designed for the quantitative inference and analysis of intercellular communication networks derived from single-cell data. Employing network analysis and pattern recognition methodologies, CellChat facilitates the prediction of principal signaling inputs and outputs of cells, elucidating the mechanisms by which these cells and signals orchestrate their functions. In this study, we employed standardized single-cell expression profiles as input data, alongside cell subtype classifications obtained through single-cell analysis, to serve as cell-specific information. We conducted an in-depth examination of cell-related interactions, quantifying the strength and frequency of cell-to-cell interactions to observe the activity and impact of each cell type in the disease.

### 2.6 Feature selection process of LASSO regression and SVM algorithm

We utilized the Least Absolute Shrinkage and Selection Operator (LASSO) logistic regression and Support Vector Machine (SVM) algorithms to select features for diagnostic markers of diseases. The LASSO algorithm utilizes the “glmnet” package, while SVM-Recursive Feature Elimination (SVM-RFE) is a machine learning method based on support vector machines (17). SVM-RFE removes feature vectors generated by SVM to identify optimal variables, and establishes a support vector machine model through the “e1071” package to further assess the diagnostic value of these biomarkers in disease contexts.

### 2.7 Immune infiltration analysis

The CIBERSORT method is a prevalent technique for assessing immune cell types within microenvironments (18). In this study, utilized the CIBERSORT algorithm was employed to analyze patient data, allowing for the inference of the relative proportions of 22 immune-infiltrating cell types. Furthermore, a correlation analysis was conducted to examine the relationship between gene expression and immune cell content.

### 2.8 GSEA analysis

Patients were categorized into high and low-expression groups based on the expression of key genes. Subsequently, Gene Set Enrichment Analysis (GSEA) was utilized to examine disparities in signaling pathways between these cohorts. The annotation gene set employed for the subtype pathway analysis was derived from version 7.0 of the Molecular Signatures Database (MsigDB). Differential expression analysis of pathways between the groups was conducted, and significantly enriched gene sets (adjusted *p-value* < 0.05) were ranked by consistency score. GSEA is frequently used to explore the correlation between disease classification and biological significance.

### 2.9 GSVA analysis

Gene Set Variation Analysis (GSVA) is a nonparametric, unsupervised method for assessing gene set enrichment in transcriptome data. GSVA assigns a comprehensive score to each gene set of interest, converting gene-level changes into pathway-level changes. This allows for the identification of potential biological function changes in different samples. In this study, gene sets were downloaded from MsigDB, and the GSVA algorithm was applied to comprehensively score each gene set, enabling the evaluation of potential biological function differences among the samples.

### 2.10 Non-coding RNA network associated with key genes

MicroRNAs (miRNAs) are small non-coding RNAs known to regulate gene expression by facilitating mRNA degradation or inhibiting mRNA translation. Consequently, we conducted an in-depth analysis to determine the presence of miRNAs associated with key genes involved in the transcriptional regulation or degradation of potentially deleterious genes. We identified miRNAs related to these key genes using the miRcode database and subsequently visualized the miRNA-gene interaction network utilizing Cytoscape software (19).

### 2.11 Transcription factor regulatory network

This study utilized the R package “RcisTarget” to predict transcription factors, with all computations conducted by RcisTarget being predicated on motif analysis. The normalized enrichment score (NES) of a motif depended on the total number of motifs in the database. In addition to the motifs annotated by the source data, we inferred further annotation files based on motif similarity and gene sequences. To estimate the overrepresentation of each motif in the gene set, we initially calculated the area under the curve (AUC) for each pair of motif-motif set. This was performed based on the recovery curve calculation of the gene set ranking of the motifs. The NES of each motif was calculated based on the AUC distribution of all motifs in the gene set.

### 2.12 Source of human sample

To verify the expression of target genes in the diseased colon tissue of UC patients, tissue biopsy samples were collected from UC patients within the research cohort at the Digestive Endoscopy Center of Changshu Hospital Affiliated to Nanjing University of Chinese Medicine (Ethical Number: CZYLS-2024120). Patients with UC secondary to other diseases or with differing pathological results were excluded. Normal tissue samples for the control group were obtained from the periphery of pathological specimens diagnosed with colon cancer and subjected to Miles surgery in the General Surgery Department of Changshu Hospital Affiliated to Nanjing University of Chinese Medicine. The collection of all samples was approved by the hospital’s Ethics committee, and written informed consent was obtained from the patients.

### 2.13 Immunohistochemistry

Colon tissue sections fixed with paraformaldehyde were deparaffinized using xylene and incubated with primary antibodies (tissue inhibitor of metalloproteinase 1 (*TIMP1*):1:200, Absin, Shanghai, China; G protein subunit gamma 5 (*GNG5*):1:200, Abcam, Shanghai, China) at 37°C for 1.5 hours. After three washes with PBS, immunocomplex detection was performed using diaminobenzidine, and nuclei were counterstained with hematoxylin. The sections were examined under a microscope (Leica, Wetzlar, Germany) (20).

### 2.14 Animals and treatment

Male C57BL/6 mice (weighing 18–20 grams) were obtained from Beijing Vital River Laboratory Animal Technology Co., Ltd. (SCXK-2021-0011, Beijing, China). Before the experiments, the mice were provided with standard laboratory chow and water ad libitum under controlled conditions of 60 ± 5% humidity, 23 ± 1°C temperature, and a 12-hour light/dark cycle. The experimental protocol was approved by the Ethics Committee of the Experimental Animal Center at Nanjing University of Chinese Medicine (Ethical Number: NJUCCSHAE-2021-1123). The mice were randomly divided into two groups: a control group and a dextran sulfate sodium (DSS) group, each with 6 mice. The control group received drinking water, while the DSS group was given 3% DSS in drinking water for 7 days (21). On the 8th day, all mice were euthanized, and their colon samples were collected for further analysis.

### 2.15 Hematoxylin and eosin staining

Colon tissue was fixed in 4% paraformaldehyde, subsequently embedded in dehydrated paraffin, and sectioned at a thickness of 4.5μm. The sections were then stained with H&E. Pathological alterations in the tissue samples were examined using an optical microscope (Leica, Wetzlar, Germany).

### 2.16 Enzyme-linked immunosorbent assay

Accurately weigh the colon tissue to achieve a weight (mg) to volume (µL) ratio of 1:9. Add nine times the volume of physiological saline and homogenize the mixture mechanically under ice water bath conditions to prepare a 10% homogenate. Centrifuge the homogenate at 2500–3000 rpm for 10 minutes and collect the supernatant for subsequent ELISA analysis. Following the manufacturer’s protocol, the concentrations of TNF-α (mIC50536-1, Mlbio, Shanghai, China) and interleukin-6 (IL-6) (ml098430, Mlbio, Shanghai, China), were quantified using a commercially available ELISA kit.

### 2.17 Immunofluorescence staining for co-localization validation

The sample slices were fixed in 10% formalin, embedded in paraffin, dewaxed, and subjected to antigen retrieval. After a one-hour blocking step at room temperature, the slices were incubated overnight at 4°C with primary antibodies *TIMP1* (1:200, Absin, Shanghai, China) and F4/80 (1:50, Abcam, Shanghai, China). Following three 10-minute washes with PBS, the slices were incubated for one hour at room temperature with Alexa Fluor 488 and Alexa Fluor 594 secondary antibodies. After another three PBS washes, an anti-quenching medium was used to mount the cover glass onto the slide. The sections were then examined under a fluorescence microscope (Leica, Wetzlar, Germany) at a magnification of 80 for microscopic analysis and imaging (22).

### 2.18 Western blot for expression validation

Total protein was extracted from colon tissue samples of human or mouse origin using RIPA lysis buffer (Beyotime, Nanjing, China) and quantified using the Bicinchoninic Acid (BCA) protein assay kit (Beyotime, Nanjing, China). Subsequently, 20 micrograms of protein were separated on a 10% SDS-PAGE gel and transferred to a polyvinylidene fluoride (PVDF) membrane. The membrane was then blocked with 5% (w/v) bovine serum albumin (BSA) or skim milk at room temperature for 1 hour. Following the blocking step, the membrane was incubated overnight at 4°C with primary antibodies targeting *GNG5* (1:1000, Absin, Shanghai, China), *TIMP1* (1:1000, Absin, Shanghai, China), and GAPDH (1:5000, Proteintech, Wuhan, China). On the next day, the membrane was incubated with secondary antibodies (horseradish peroxidase-conjugated goat anti-rabbit or anti-mouse IgG, 1:5000, Cell Signaling Technology, Danvers, MA, USA) at room temperature for 1 hour. Visualization of the protein bands was performed using an ECL detection kit and a gel imaging system (Tanon, Shanghai, China). The intensity of the bands was then quantified using the densitometric analysis feature of Gel Pro 4.0 software (Tanon, Shanghai, China) (23).

### 2.19 Statistical analysis

All statistical analyses were performed using the R programming language (version 4.3.0), with a significance threshold set at *p* < 0.05.

## 3 Results

### 3.1 Preliminary processing of single-cell expression profile data

During the initial processing of single-cell expression profile data, rigorous adherence to established quality control and standardized procedures was maintained. Following the screening process, cells with fewer than 200 captured genes were excluded, while those meeting the criteria were retained based on indicators such as nFeature-RNA, nCount-RNA, and percent.mt, resulting in a dataset of 22,345 high-quality cells. Concurrently, doublets were removed, and the top 2,000 highly variable genes were selected for subsequent analysis. The processed data demonstrated favorable distribution characteristics, as evidenced by violin plots and scatter plots, thereby establishing a robust foundation for precise cell subpopulation annotation and gene expression analysis in future studies (**Supplementary Figure S1A, C**). This approach effectively mitigates analysis bias associated with data quality issues.

### 3.2 Single-cell data cell subpopulation annotation and ligand-receptor interaction analysis (Cellchat)

**Figure 2:**
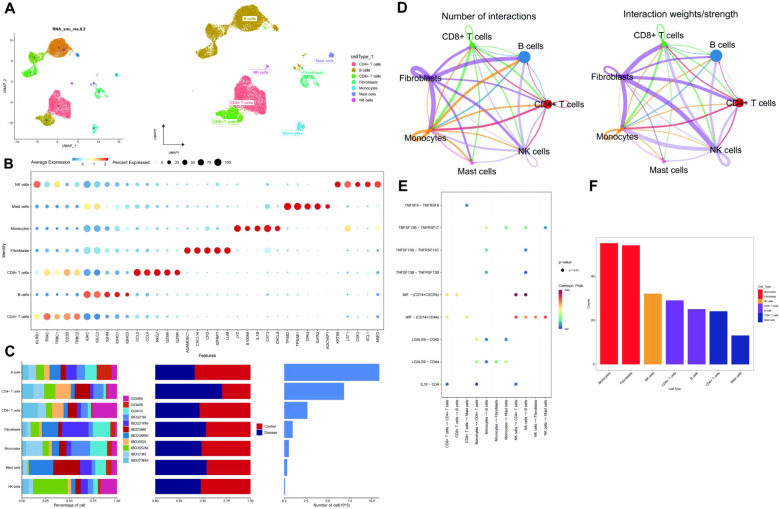
Cell annotation and communication. **(A)** UMAP-based clustering of cells into 12 groups. Classification of clusters into 7 cell types: CD4^+^ T cells, B cells, CD8^+^ T cells, Fibroblasts, Monocytes, Mast cells, and NK cells. **(B)** A Dotplot visualization of cell type markers and their expression levels. **(C)** Bar charts displaying the proportions and content of 7 cell types in the sample. **(D)** Cell interaction network among 7 cell types based on communication probability and strength. **(E)** Bubble plot of receptor-ligand interactions between cells, with colors showing communication probabilities. **(F)** Comparison of total interactions among 7 cell types, showing a decreasing trend from left to right, with Monocytes having the strongest interactions.

The data underwent standardization, homogenization, and subsequent analysis using Principal Component Analysis (PCA), Harmony, and Uniform Manifold Approximation and Projection (UMAP) (**Supplementary Figures S1D-F**, **Figure 2A**). Each subtype was annotated to one of seven cell categories: CD4^+^ T cells, B cells, CD8^+^ T cells, Fibroblasts, Monocytes, Mast cells, and NK cells (**Figure 2A**). A bubble plot and histogram were generated to visualize the expression of classic markers and cell proportions for these categories (**Figures 2B, C**). The software package Cellchat was employed to examine ligand-receptor interactions within the single-cell expression profile, revealing intricate relationships between the cell subtypes (**Figure 2D**). Notably, Monocytes demonstrated a closer potential interaction with other cell types (**Figures 2E, F**).

### 3.3 Feature selection process of LASSO regression and SVM algorithm

**Figure 3:**
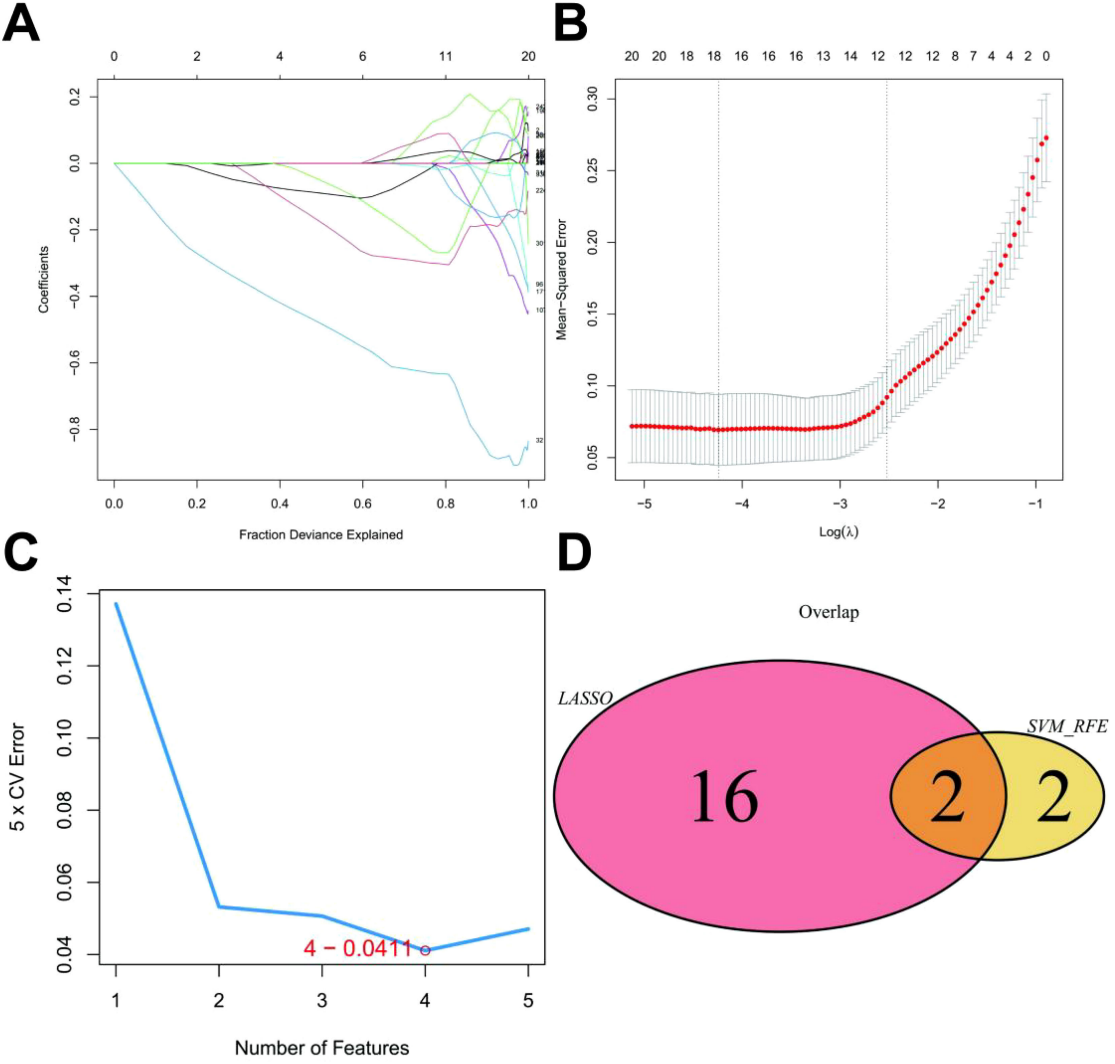
LASSO Model Construction. **(A)** LASSO coefficient distribution and gene combination at the minimum lambda value. **(B)** Ten-fold cross-validation for tuning parameter selection to find the minimum lambda. **(C)** Top four feature genes with the lowest error rate in the SVM algorithm. **(D)** Venn plot showing two overlapping genes selected by both LASSO regression and SVM algorithms.

To investigate the genetic underpinnings of UC, we retrieved expression profile data from the GEO database (GSE48958). This dataset comprised 21 patient samples. To identify key genes associated with this condition, we employed a two-step approach. First, we utilized a combination of LASSO regression and SVM algorithms to screen the 386 monocyte marker genes (*p_adj* < 0.05 & *LogFC* > 0.585) previously identified. LASSO regression yielded 18 characteristic genes (**Figures 3A, B**), while SVM identified 4 (**Figure 3C**). By intersecting these gene sets, we identified 2 genes, *GNG5* and *TIMP1*, as the most promising candidates for further exploration in our research on UC (**Figure 3D**).

### 3.4 Immune infiltration analysis

**Figure 4:**
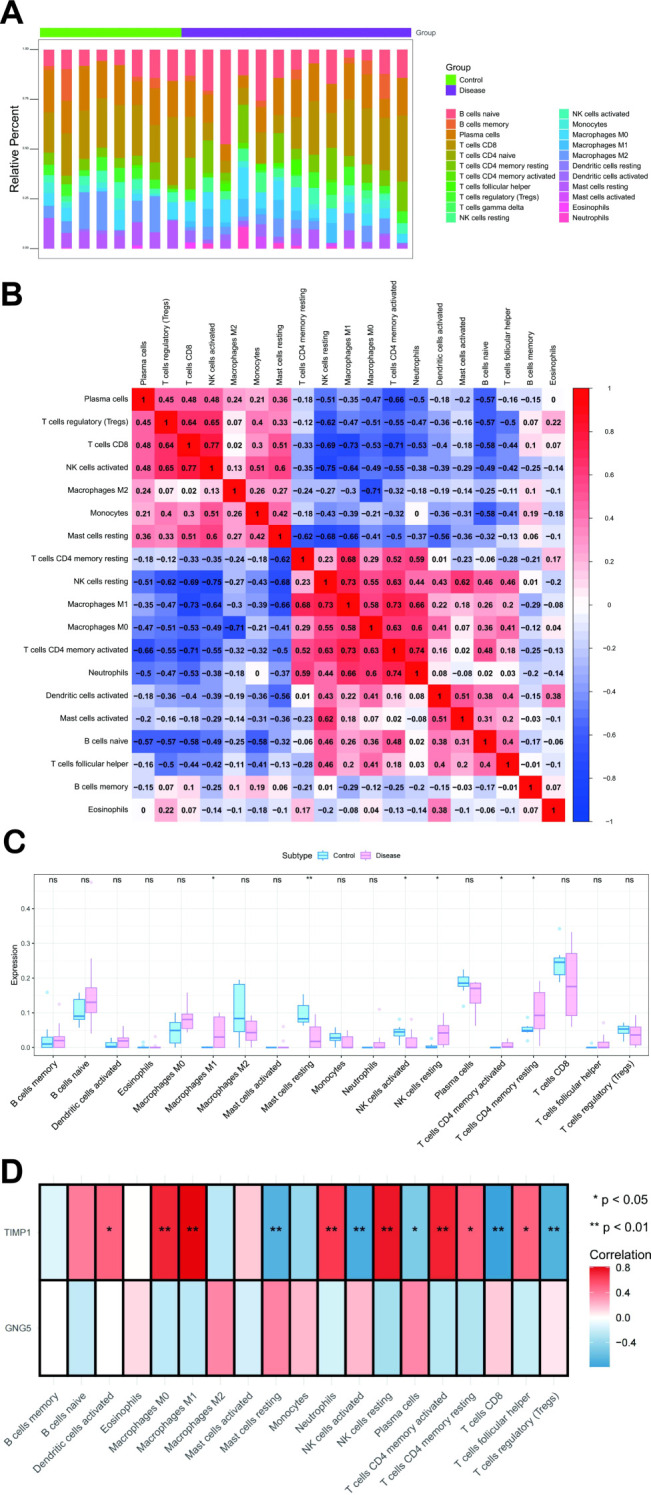
Immune Infiltration Analysis. **(A)** Comparison of immune cell subset percentages between control and disease groups, with immune cells on the x-axis and relative percentages on the y-axis. **(B)** Correlation of immune cell infiltration, showing cell types on both axes; red indicates positive correlation, blue indicates negative, and darker colors signify stronger associations. **(C)** Blue and pink bars show the immune cell content differences between control and disease groups, respectively, with cell types on the x-axis, scores on the y-axis, and * indicating statistical significance. **(D)** The x-axis shows immune cell types, the y-axis shows two key genes, and an asterisk marks statistical significance in their correlation. **: p < 0.01.

The microenvironment, a pivotal factor in disease progression, consists of a complex interplay between cellular and extracellular components. This intricate ecosystem includes fibroblasts, immune cells, extracellular matrix, growth factors, inflammatory factors, and unique physical and chemical properties. The microenvironment exerts a substantial influence on disease diagnosis, prognosis, and therapeutic response. Our investigation revealed distinct patterns of immune cell infiltration and correlation in various disease states (**Figures 4A, B**). Compared to the control group, the disease group exhibited significantly elevated levels of M1 Macrophages, resting NK cells, CD4^+^ memory activated T cells, and CD4^+^ memory resting T cells. Conversely, resting Mast cells and NK cells activated were significantly reduced in the disease group (**Figure 4C**). Further analysis of the relationship between key genes and immune cells demonstrated a strong positive correlation between *TIMP1* and several immune cell types, including CD4^+^ memory resting T cells, CD4^+^ memory activated T cells, follicular helper T cells, resting NK cells, M0 Macrophages, M1 Macrophages, activated Dendritic cells, and Neutrophils. Conversely, *TIMP1* was negatively correlated with Plasma cells, CD8^+^,T cells regulatory T cells (Tregs), activated NK cells, and resting Mast cells (**Figure 4D**). Moreover, our analysis of the correlation between key genes and different immune factors, including immunosuppressive factors, immunostimulatory factors, chemokines, and receptors, suggests that these genes are intimately involved in shaping the immune microenvironment (**Supplementary Figures S2A-E**).

### 3.5 Signaling pathways involved in key genes

**Figure 5:**
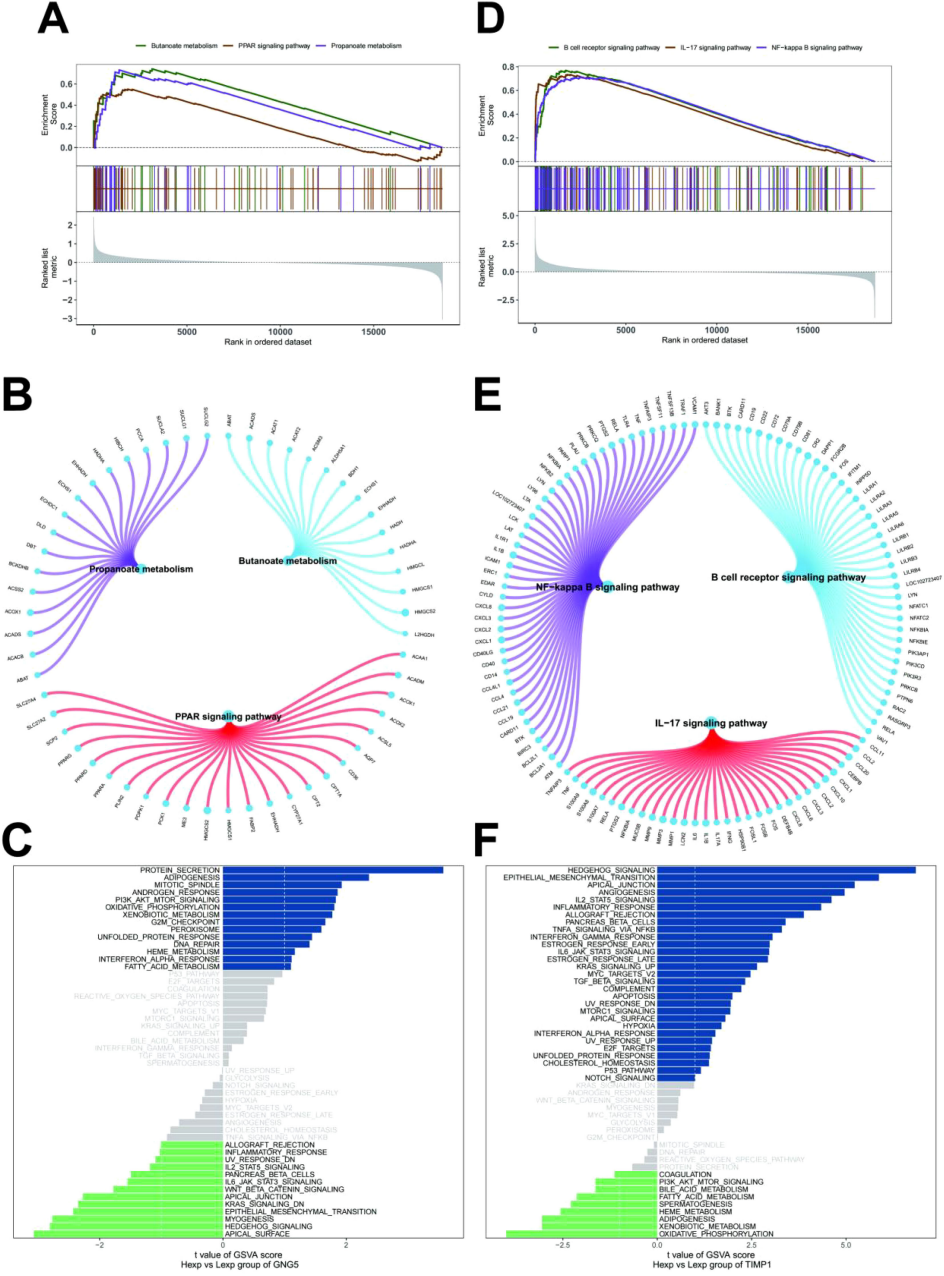
Signal pathways of key genes. **(A, B)** *GNG5* in the KEGG signaling pathway and their regulatory roles. **(C)** GSVA analysis showing signaling pathways for high expression *GNG5* (blue) and low expression *GNG5* (green), using the Hallmark gene set as a reference. **(D, E)** *TIMP1* in the KEGG signaling pathway and their regulatory roles. **(F)** GSVA analysis showing signaling pathways for high expression *TIMP1* (blue) and low expression *TIMP1* (green), using the Hallmark gene set as a reference.

To elucidate the specific signaling pathways involved in the key genes and explore their potential molecular mechanisms in disease progression, we conducted a comprehensive analysis. GSEA revealed that *GNG5* was significantly enriched in signaling pathways such as propanoate metabolism, butanoate metabolism, and peroxisome proliferator-activated receptor (PPAR) signaling (**Figures 5A, B**). *TIMP1*, on the other hand, was enriched in pathways including B cell receptor signaling, interleukin-17 (IL-17) signaling, and NF-κB signaling (**Figures 5D, E**). Additionally, GSVA identified *GNG5* as being enriched in pathways associated with protein secretion and adipogenesis (**Figure 5C**). *TIMP1* was found to be enriched in pathways related to hedgehog signaling and epithelial-mesenchymal transition (**Figure 5F**). These findings collectively suggest that the key genes may influence disease progression through these identified signaling pathways.

### 3.6 Non-coding RNA network and transcriptional regulatory network related to key genes

Subsequently, we employed the miRcode database to conduct a reverse prediction of the key genes, resulting in the identification of 20 miRNAs and a total of 23 mRNA-miRNA regulatory relationships. These interactions were visualized using Cytoscape (**Supplementary Figure S3A**). By utilizing the key genes as a gene set for this analysis, we discovered that these genes were subject to regulation by common mechanisms, such as multiple transcription factors. To identify these transcription factors, we employed cumulative recovery curves and conducted motif-transcription factor annotation and selection analysis on the key genes. The motif with the highest standardized enrichment score (NES: 14) was determined to be cisbp:M6056. We have provided a comprehensive visualization of all the enriched motifs and their corresponding transcription factors associated with the key genes (**Supplementary Figure S3B, C**).

### 3.7 Relationship between key genes and disease-related genes

**Figure 6:**
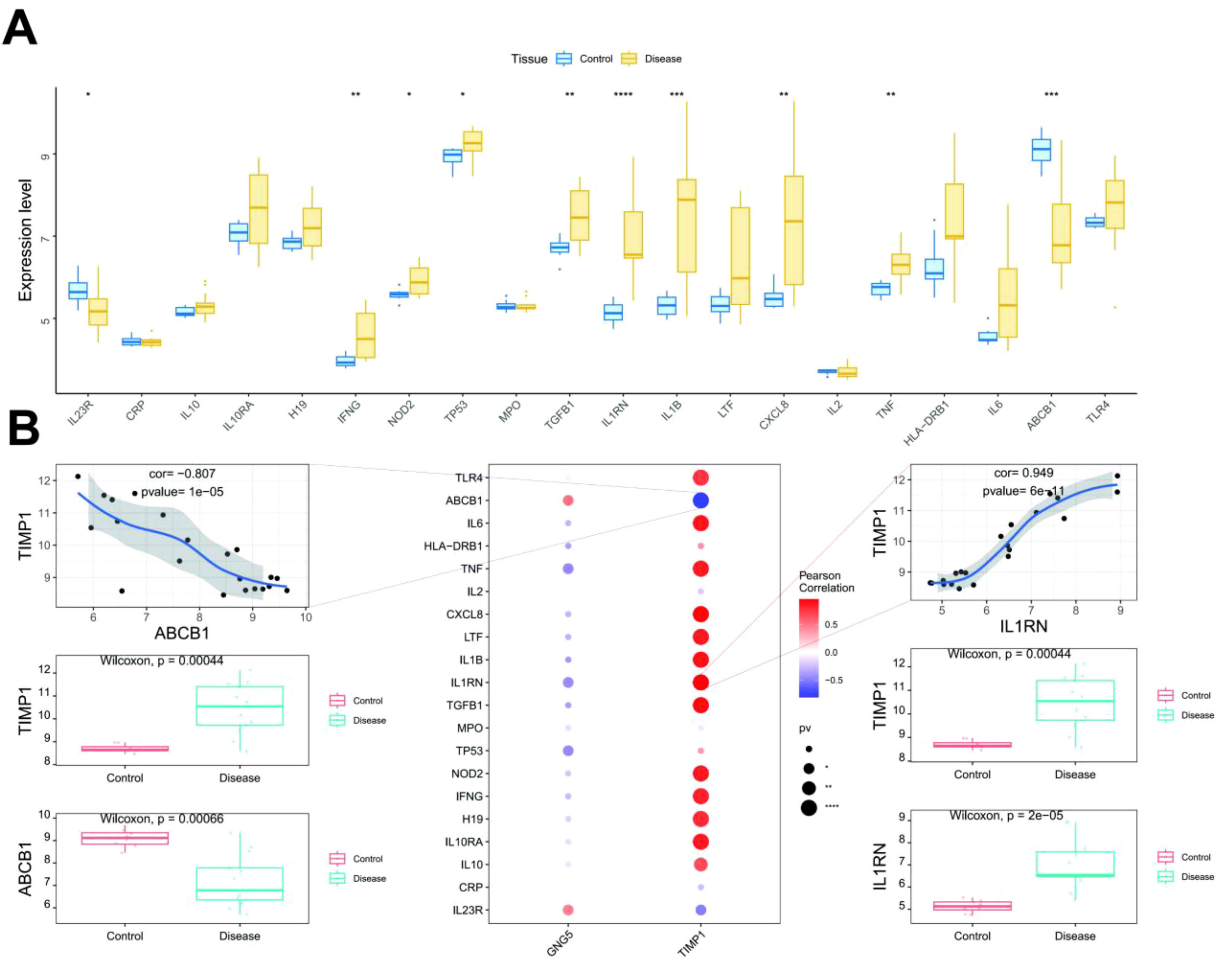
Correlation between key genes and disease genes. **(A)** The top figure illustrates the differential expression of disease regulatory genes, with blue for control patients and yellow for disease patients. **(B)** The bottom figure presents correlation analysis, where blue denotes negative and red denotes positive correlations. *: p < 0.05; **: p < 0.01; ***: p < 0.001; ****: p < 0.0001.

In the current study, the GeneCards database (https://www.genecards.org/) was utilized to identify genes potentially implicated in disease regulation. To assess inter-group expression differences amongst these genes, we analyzed the expression levels of 20 highly ranked genes (based on the Relevance*score) with confirmed expression within the transcriptome data. This analysis revealed significant expression differences between the two patient groups for genes including interleukin 23 receptor (*IL23R*), interferon gamma (*IFNG*), nucleotide-binding oligomerization domain 2 (*NOD2*), tumor protein 53 (*TP53*), transforming growth factor beta 1 (*TGFB1*), interleukin 1 receptor antagonist (*IL1RN*), interleukin-1 beta (*IL1B*), interleukin 8 (*CXCL8*), tumor necrosis factor (*TNF*) and ATP-binding cassette subfamily B member 1 (*ABCB1*) (**Figure 6A**). Furthermore, a correlation analysis was performed to investigate the relationship between key genes and disease regulation genes. The expression levels of these key genes demonstrated statistically significant correlations with the expression levels of disease regulation genes. Notably, *TIMP1* exhibited a strong positive correlation (cor = 0.949) with *IL1RN* while displaying a significant negative correlation (cor = -0.807) with *ABCB1* (**Figure 6B**).

### 3.8 Expression profile of key genes in spatial transcriptome and validation of pathological tissues derived from human sources

**Figure 7:**
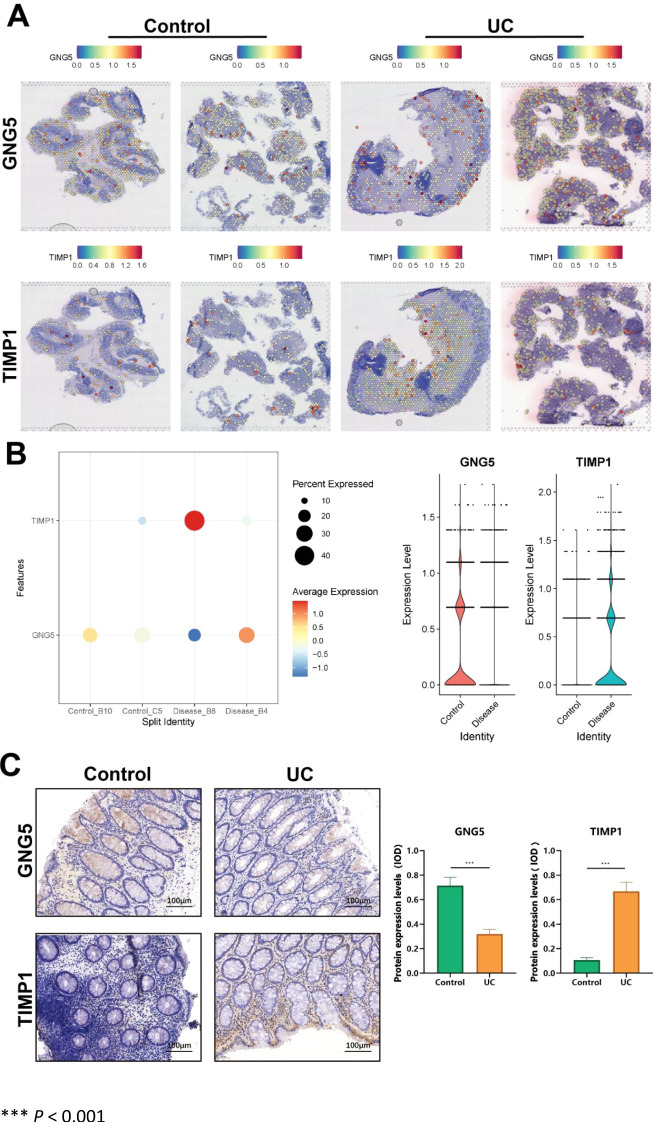
Key gene expression in spatial transcriptome and lesion tissues of UC patients. **(A)** Scatter plot showing key gene expression levels in single-cell idling. **(B)** Up: Bubble plot depicting key gene expression levels (blue = low, red = high). Down: Violin plot illustrating key gene expression distribution in single-cell idling. **(C)** Differential expression of *GNG5* and *TIMP1* between control and UC groups. *** *P* < 0.001.

We analyzed the spatial transcriptome data to assess the expression levels of two key genes. Compared with the control group, *GNG5* expression was inhibited in the disease group, while *TIMP1* expression was significantly upregulated in the disease group (**Figure 7A**). We assessed the differential expression levels of key genes across various groups utilizing bubble and violin plot visualizations. Our analysis revealed a downregulation of *GNG5* and an upregulation of *TIMP1* in UC (**Figure 7B**). IHC analysis was conducted on colon lesions from patients with UC to assess the expression levels of *GNG5* and *TIMP1*. Results demonstrated a significant upregulation of *TIMP1*, and a significant downregulation of *GNG5* in the disease group compared to the control group (*p* < 0.001), aligning with the previously presented spatial transcriptome data (**Figure 7C**).

### 3.9 Validation of key genes in tissues from a DSS-induced colitis mouse model

**Figure 8:**
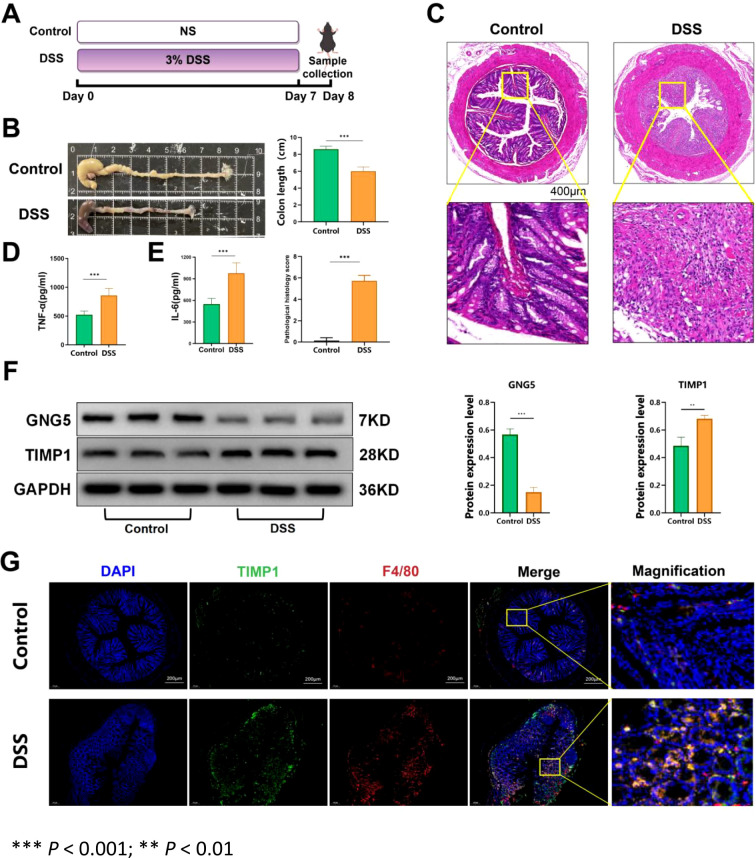
Key gene expression in DSS-induced colonic lesions in mice model. **(A)** The animal experimental protocol. **(B)** H&E staining images of colon tissue from the indicated groups. **(C)** Comparison of colon length in the indicated groups. **(D, E)** The levels of IL-6 and TNF-α in the colon homogenate of each group. F Protein levels of *GNG5* and *TIMP1* in the colon. **(G)** Co-localization of *TIMP1* with the macrophage marker F4/80 in the colon. *** *P* < 0.001; ** *P* < 0.01.

A DSS-induced colitis mice model was generated successfully (**Figure 8A**). The distal colon tissue was collected and its length was measured and then imaged. It was found that compared with the control group, the colon of the DSS-induced colitis mice model was significantly shortened under inflammatory stimulation (*p* < 0.001) (**Figure 8B**). The colon tissue of mice was collected for H&E staining. As illustrated in **Figure 8C**, DSS induction resulted in the desquamation and necrosis of colonic epithelial cells, infiltration of inflammatory cells within the mucosal layer, and loss of crypt structures in DSS-induced colitis model mice. The levels of IL-6 and TNF-α in colon tissue were measured utilizing ELISA. The results revealed a significant increase in the levels of inflammatory cytokines IL-6 and TNF-α in the colon tissue of DSS-induced colitis model mice (*p* < 0.001) compared with the control group (**Figures 8D, E**). To further elucidate the expression of key genes in UC, we conducted Western blot to evaluate corresponding protein expression levels in colon samples obtained from a DSS-induced colitis mouse model. Results indicated that, compared to the control group, *GNG5* protein levels were significantly downregulated and *TIMP1* levels were significantly upregulated in the DSS group (*p* < 0.001, *p* < 0.01), which confirmed our spatial transcriptome predictions (**Figure 8G**).

Our single-cell sequencing data showed high monocyte expression in UC samples, with macrophages being crucial monocyte components. Lots of references indicates that macrophages are vital in UC inflammation and tissue repair (24). Immune infiltration analysis revealed a stronger correlation between *TIMP1* and UC-related immune cells compared to *GNG5*. Thus, we used immunofluorescence co-localization to assess *TIMP1* expression in macrophages. The results indicated that *TIMP1* co-localizes with macrophage marker F4/80 in colon tissue, suggesting that *TIMP1* may affect disease progression through functional expression in UC colon macrophages (**Figure 8G**).

### 3.10 The link between key genes and immune metabolic pathways along with T cell exhaustion correlation analysis

**Figure 9:**
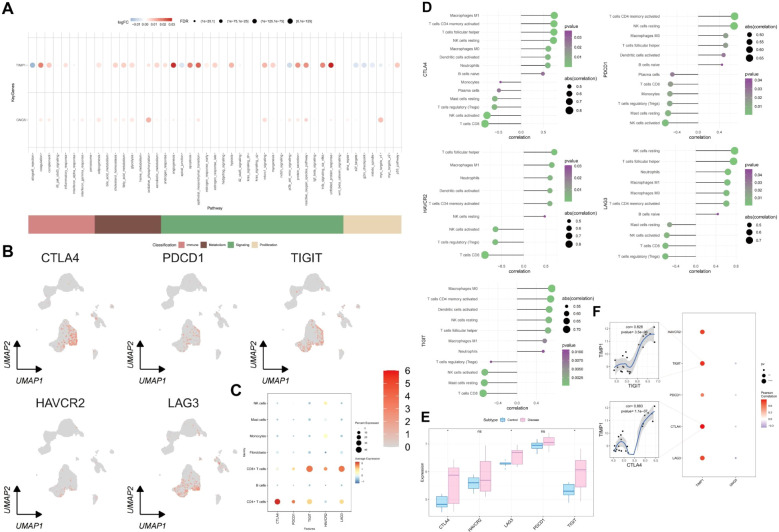
Link between key genes and immune metabolic pathways, and T cell exhaustion analysis. **(A)** Correlation bubble plot: x-axis shows immune metabolism pathways, y-axis shows two key genes, blue indicates low expression, red indicates high expression. **(B)** UMAP diagram: expression profile of exhaustion-related genes in single cells. **(C)** Bubble plot showing single-cell expression of exhausted phase genes, with blue for low and red for high expression. **(D)** Circle size and color represent the correlation coefficient and p-value between exhaustion-related genes and immune cells. **(E)** Differential expression of exhausted genes in UC. **(F)** Blue and red circles indicate negative and positive correlations between key and exhaustion-related genes, with circle size showing statistical significance.

To quantitatively assess the activity of immune metabolism genes in individual cells, we utilized AUCell. Bubble plots were employed to visualize the differential activity of key genes within these pathways. Our findings revealed that *GNG5* and *TIMP1* were significantly upregulated in oxidative phosphorylation, the unfolded protein response, and related pathways (**Figure 9A**). Furthermore, an analysis of classical exhaustion-related genes (*LAG3*, *PDCD1*, *TIGIT*, *HAVCR2*, *CTLA4*) in single cells indicated a pronounced T cell exhaustion phenotype (**Figures 9B, C**). We also investigated the correlation between exhaustion-related genes and immune infiltration as well as their differential expression in the transcriptome. *CTLA4*, *LAG3* and *TIGIT* were found to be significantly up-regulated in UC (**Figure 9D**).Our analysis identified a significant positive correlation between five exhaustion-related genes and activated memory CD4^+^ T cells, T follicular helper cells, as well as other cell types. (**Figure 9E**). To delve deeper into the relationship between *GNG5*, *TIMP1*, and cellular depletion, a correlation analysis was conducted involving five depletion-related genes. This analysis identified a significant positive correlation between *TIMP1* and *TIGIT* as well as *CTLA4* (**Figure 9F**) (*p* = 3.5e-06, 1.1e-07).

## 4 Discussion

The integration of single-cell and spatial transcriptomic analyses in this study has yielded novel insights into the cellular heterogeneity and molecular dynamics underlying the pathogenesis of UC. Our findings underscore the pivotal roles of specific monocyte subtypes and two key genes, *GNG5* and *TIMP1*, in modulating the inflammatory microenvironment and driving disease progression. These discoveries not only enhance our understanding of UC immunopathology but also suggest potential therapeutic targets for precision medicine. Utilizing single-cell transcriptomics, we identified distinct subpopulations of monocytes characterized by altered communication networks in UC, and monocytes demonstrated the most robust ligand-receptor interactions. The application of machine learning techniques, specifically LASSO and SVM algorithms, further identified *GNG5* and *TIMP1* as central regulatory elements in UC. *GNG5*, a G protein subunit involved in signal transduction, was significantly downregulated in UC lesions, whereas *TIMP1*, a metalloproteinase inhibitor associated with extracellular matrix remodeling, was markedly upregulated. These findings are corroborated by spatial transcriptomic and immunohistochemical analyses in human UC tissues, as well as in DSS-induced murine colitis models, thereby confirming their consistent dysregulation across species.

In UC, the disproportionate distribution of monocyte subtypes—characterized by a predominance of classical and intermediate subtypes with pro-inflammatory tendencies, alongside functional impairments in non-classical subtypes—contributes to the pathogenesis of intestinal inflammation and fibrosis. Monocytes are capable of further differentiation into macrophages within specific tissues, including the intestinal and dermal regions (25). Classical monocytes, identified by the CD14^++^/CD16 phenotype, engage C-C motif chemokine receptor 2 (CCR2) signaling, which is pivotal for their function, thereby activating downstream NF-κB and MAPK pathways. This activation facilitates their differentiation into pro-inflammatory M1 macrophages and augments the secretion of inflammatory mediators, including interleukin-1β (IL-1β) and reactive oxygen species (ROS) (26). CCR2 signaling has the potential to enhance TIMP1 expression, potentially through the activation of the PI3K/Akt pathway, inhibit MMP-9 activity, and consequently exacerbate extracellular matrix (ECM) deposition and fibrosis (27). Intermediate monocytes, identified by the CD14^+^/CD16^+^, undergo differentiation into M2 macrophages in response to the influence of TGF-β. The GNG5 protein is involved in G protein-coupled receptor (GPCR) signaling pathways, including those mediated by CCR2 and chemokine (C-X3-C motif) receptor 1 (CX3CR1). The absence of GNG5 may impede the migration of monocytes to the intestinal environment and their subsequent differentiation into anti-inflammatory phenotypes, such as M2 macrophages (28).

*GNG5*, a member of the G protein gamma subunit family, is a component of the glutamate transporter family. G proteins are essential signaling molecules involved in various physiological processes. *GNG5* plays a regulatory role in the body, influencing cell proliferation, differentiation, and metabolism. Previous studies (29) have implicated *GNG5* in glioma cell proliferation, migration, and macrophage infiltration. *GNG5* is involved in cell cycle regulation and promotes cell proliferation, potentially through the modulation of growth factor receptor-associated signaling pathways (30). Additionally, *GNG5* has been linked to apoptosis in human chondrocytes (31) and lung cancer cells (32). However, the correlation between *GNG5*, UC, and immune cells remains understudied. In this study, we present novel findings identifying *GNG5* as a target gene associated with monocyte markers and characteristics of UC. The observed downregulation of *GNG5* in UC tissues, as evidenced by spatial transcriptomic, immunohistochemical, and murine model analyses, indicates its potential involvement in maintaining mucosal homeostasis. *GNG5* is implicated in modulating intracellular signaling pathways, including those mediated by G Protein-Coupled Receptors (GPCRs), which are essential for immune cell activation and epithelial repair. Our analyses utilizing GSEA and GSVA have linked *GNG5* to propanoate metabolism and the PPAR signaling pathways, both of which are known for their roles in regulating anti-inflammatory responses and preserving epithelial barrier integrity. The reduced expression of *GNG5* may undermine these protective mechanisms, potentially exacerbating inflammation and tissue damage.

*TIMP1* is a zinc and calcium-containing proteolytic enzyme secreted by neutrophils and lymphocytes. Its primary function is to inhibit matrix metalloproteinases (MMPs), which are crucial for extracellular matrix (ECM) degradation. MMPs are upregulated after tissue injury and are involved in cytokine activation, cell migration, and ECM remodeling. TIMPs balance MMP activity, promoting tissue wound healing (33). MMPs and TIMPs are key regulators in IBD pathogenesis. Their imbalance is associated with inflammation and intestinal fibrosis in IBD (34). MMPs also modulate the inflammatory response by cleaving and activating cytokines, intensifying inflammation (35). Despite *TIMP1*’s inhibitory effect on MMPs, it does not exhibit the expected anti-inflammatory properties in inflammatory diseases. Elevated *TIMP1* levels have been associated with poor prognosis in various inflammatory conditions (36). Schoeps et al. (37) found that high *TIMP1* expression can activate neutrophils to release neutrophil extracellular traps (NETs). In patients with IBD, *TIMP1* expression is significantly elevated in colon tissue and serum, correlating with disease severity (38). These findings suggest that *TIMP1*’s pro-inflammatory properties outweigh its MMP inhibitory effects in inflammatory diseases. In this study, we hypothesized that *TIMP1* is a target gene associated with monocyte markers and UC characteristics, with more significant interaction with immune cells than *GNG5*. *TIMP1* expression is markedly upregulated in UC samples and positively correlates with various immune cell populations, including M0 and M1 macrophages, activated dendritic cells, and neutrophils. Human sample analysis corroborated these findings. A DSS-induced acute colitis model in mice revealed a significant increase in *TIMP1* protein levels in colonic tissue compared to controls. Immunofluorescence demonstrated *TIMP1* co-localization with the macrophage marker F4/80, suggesting that *TIMP1*’s pro-inflammatory effects in mice may be mediated through its influence on macrophage function in the colon.

UC is often associated with dysregulated intestinal immune cells, particularly T cell activation and functional alterations. T cell exhaustion, a progressive decline in T cell function under prolonged immune stimulation, is common in chronic infections and tumor microenvironments. This phenomenon limits T cell antigen response capacity and effector functions, reducing overall immune response efficacy (39). Exhausted T cells exhibit decreased proliferative capacity, upregulated inhibitory receptors(programmed cell death protein 1 (*PD-1*), *CTLA4*, and *LAG3*), and loss of effector function (40). *LAG3* and *PD-1* co-expression drives T cell exhaustion and regulates the expression of thymus high mobility group box protein (TOX). Ectopic TOX expression in effector T cells induces T cell exhaustion transcriptional programs (41). Slevin et al. (42) reported *LAG3* upregulation in mucositis, primarily on effector memory T cells, correlating with disease activity, suggesting *LAG3* as a potential therapeutic target for UC. However, a clinical trial by D’Haens et al. (43) showed that *LAG3*-depleting monoclonal antibody GSK2831781 did not reduce colonic mucosal inflammation despite successful *LAG3* depletion. Therefore, the mechanisms linking T cell exhaustion to UC prognosis require further exploration. Our analysis of immune infiltration has identified a skewed microenvironment in UC, characterized by a predominance of M1 Macrophages, activated CD4^+^ T cells, and Neutrophils, alongside a reduction in regulatory T cells and resting Mast cells. This imbalance reflects the pro-inflammatory environment observed in advanced stages of UC, where persistent inflammation contributes to tissue damage. Importantly, *TIMP1* was identified as a central gene positively associated with markers of T cell exhaustion, such as *TIGIT* and *CTLA4*. T cell exhaustion, indicative of chronic antigen exposure, is increasingly recognized in UC and may account for the limited effectiveness of current immunotherapies. The co-enrichment of *TIMP1* with the IL-17 and NF-κB pathways further implicates it in sustaining Th17-driven inflammation, a critical pathway in UC pathogenesis. These findings suggest that targeting *TIMP1* or its downstream effectors could alleviate both inflammation and immune exhaustion, providing a dual therapeutic approach.

## 5 Conclusion

This study represents the inaugural integration of single-cell and spatial transcriptomics methodologies to examine the spatial distribution characteristics of cellular heterogeneity and gene expression in UC. The investigation elucidates the metabolic regulatory role of *GNG5* and the pro-inflammatory and depletion-promoting effects of *TIMP1*, identifying them as potential novel biomarkers or targets for therapeutic intervention. The study further identified that *TIMP1* facilitates the progression of ulcerative colitis via a dual mechanism involving T cell depletion and macrophage activation. This finding offers a theoretical foundation for developing therapeutic strategies aimed at targeting *TIMP1* or other associated immune checkpoints.

## Data Availability

The original contributions presented in the study are included in the article/**Supplementary Material**. Further inquiries can be directed to the corresponding authors.
