# Supplementary material for: Investigating the molecular mechanisms associated with ulcerative colitis through the application of single-cell combined spatial transcriptome sequencing
Source: Front Immunol. 2025 May 13;16:1534768. doi: 10.3389/fimmu.2025.1534768 (PMC12106440; doi:10.3389/fimmu.2025.1534768)
Supplement: Supplementary file 1 [file DataSheet1.docx]

**
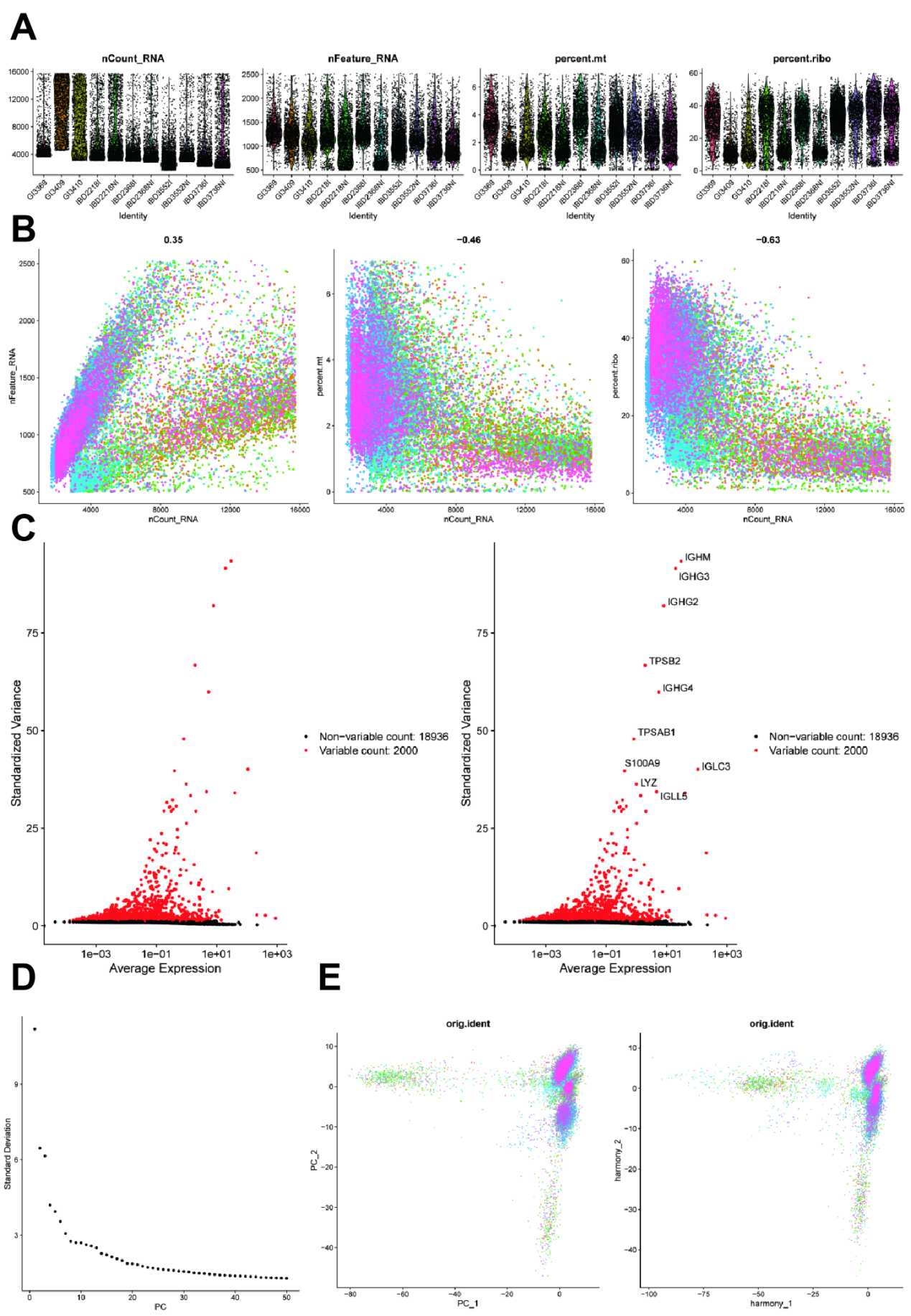
**

**Supplementary material 1**

**
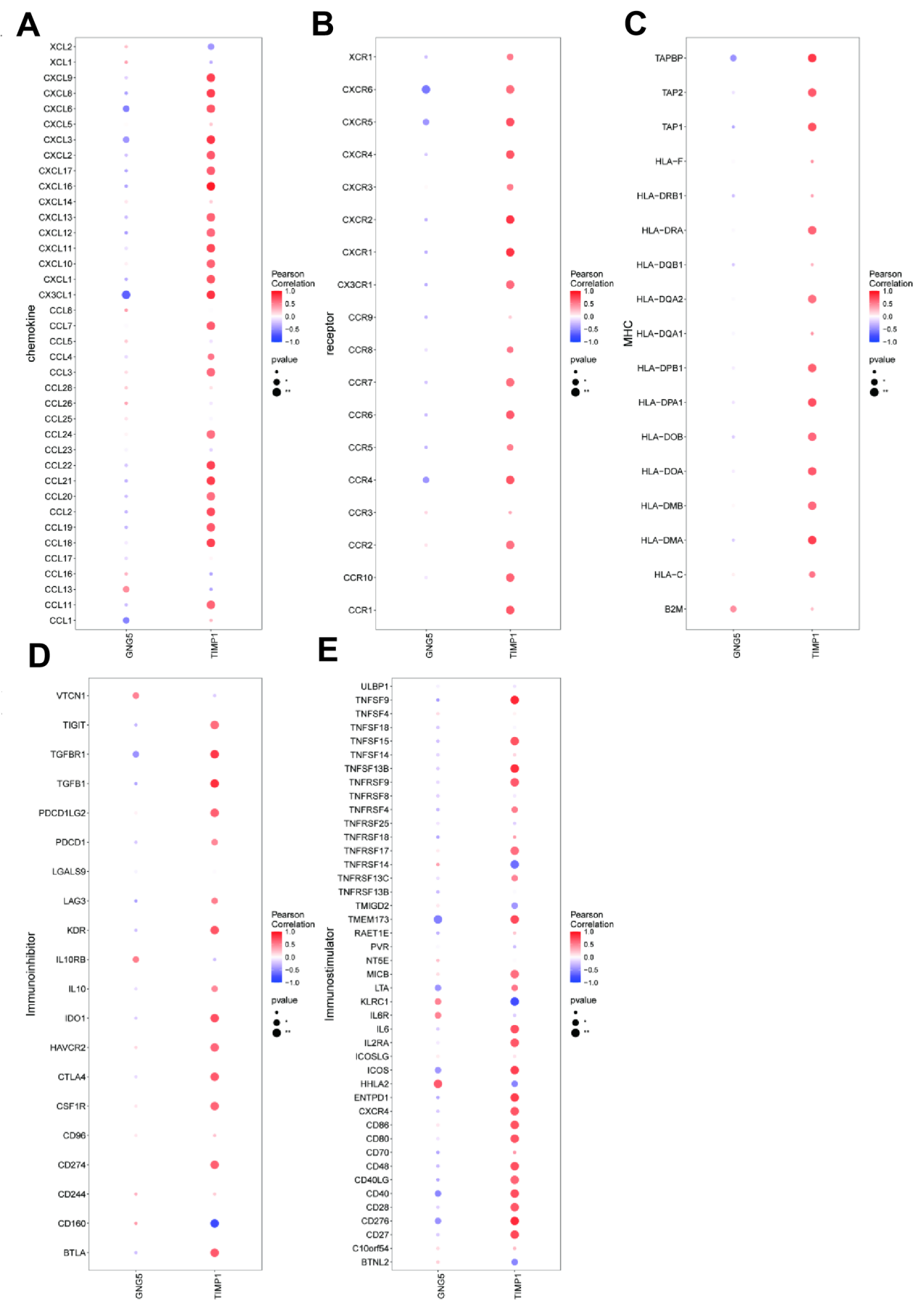
**

**Supplementary material 2**

**
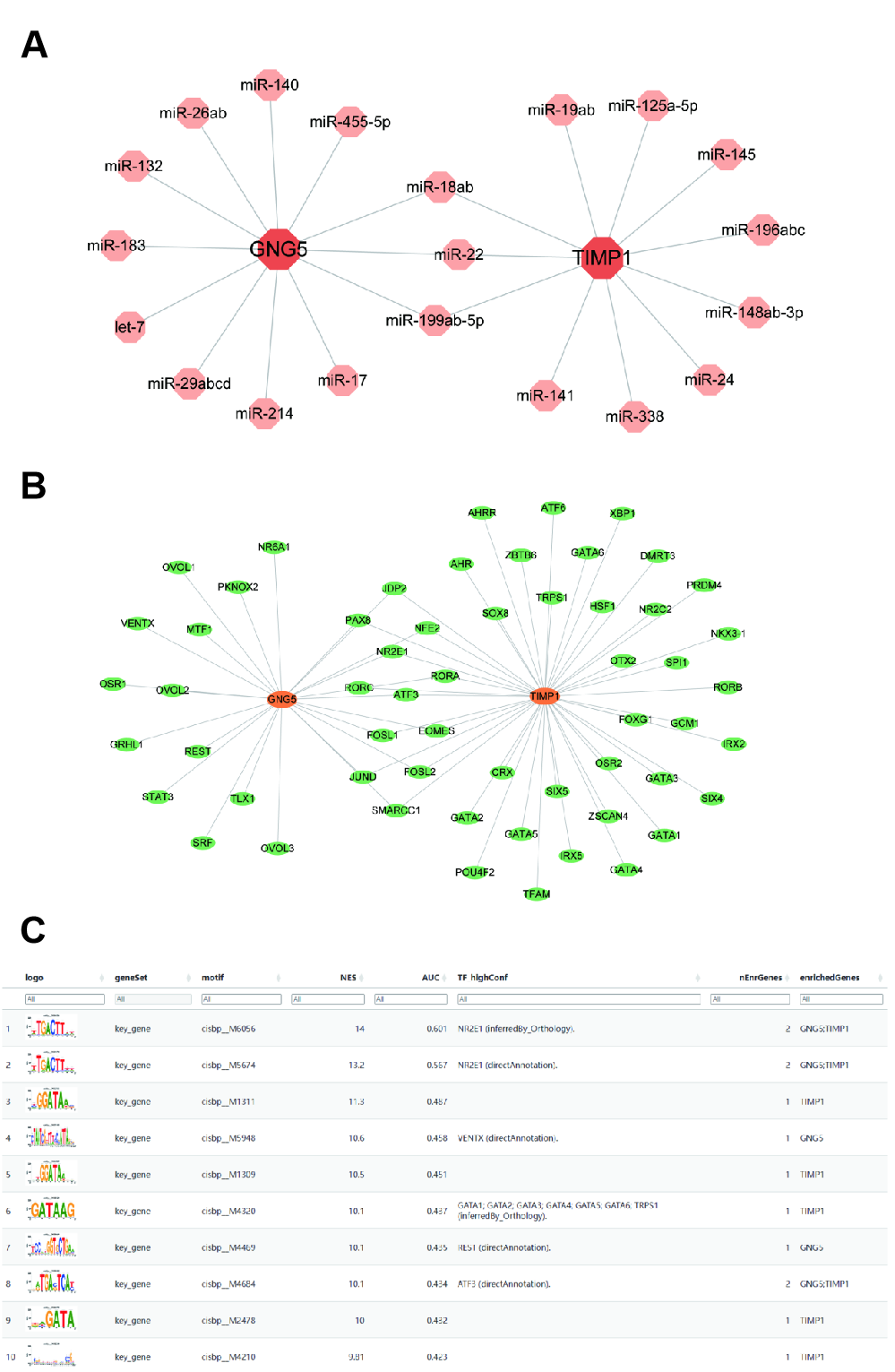
**

**Supplementary material 3**

**
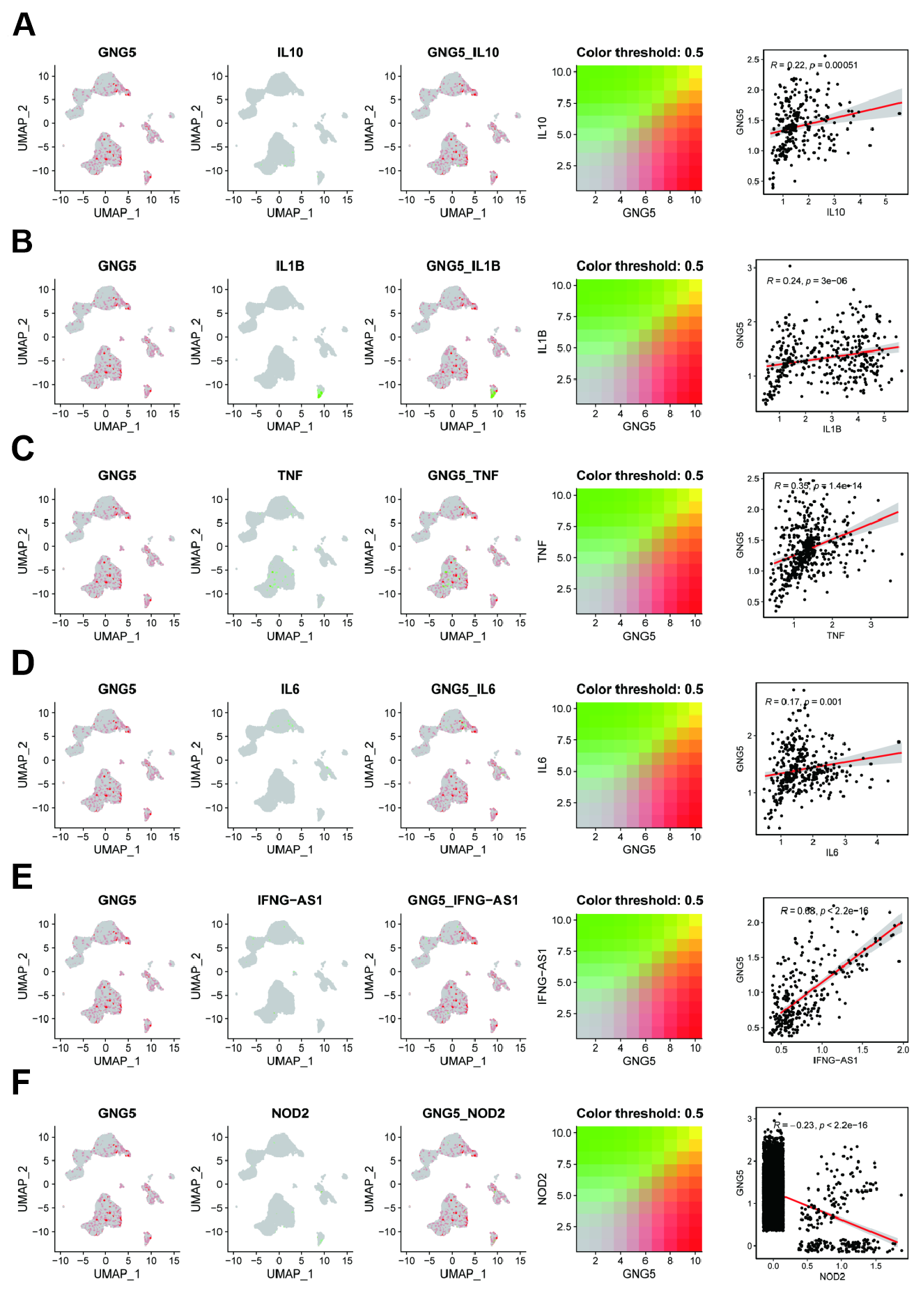
**

**Supplementary material 4-1**

**
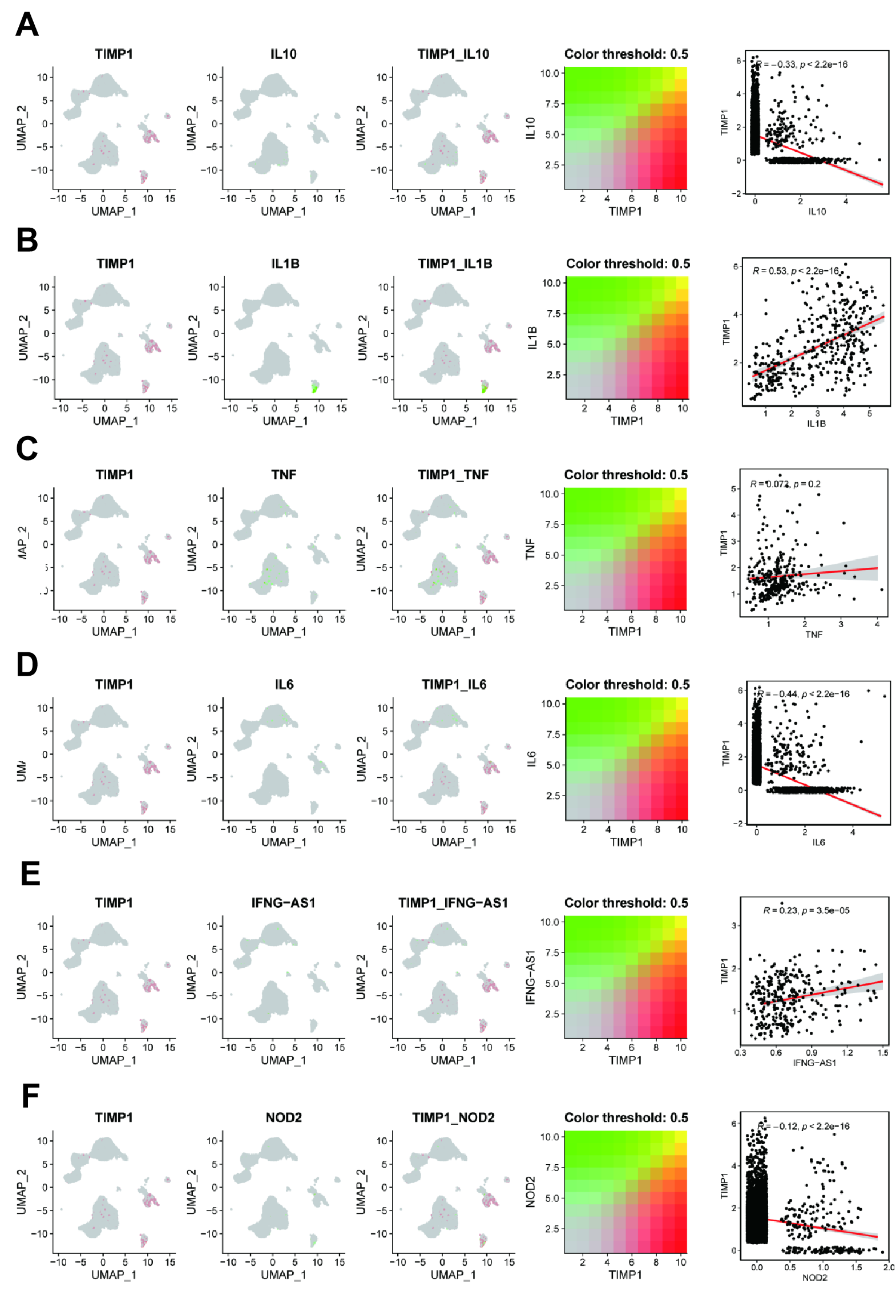
**

**Supplementary material 4-2**
